# Atherosclerosis: From Molecular Biology to Therapeutic Perspective

**DOI:** 10.3390/ijms23073444

**Published:** 2022-03-22

**Authors:** Ida Perrotta

**Affiliations:** Centre for Microscopy and Microanalysis (CM2), Department of Biology, Ecology and Earth Sciences, University of Calabria, Arcavacata di Rende, 87036 Cosenza, Italy; idaperrotta@yahoo.it

Atherosclerosis is a chronic and progressive inflammatory disease of the arteries initiated by the functional and structural alteration of the endothelial layer responsible for promoting the subendothelial retention of modified low-density lipoproteins (LDL), which in turn generate an active proinflammatory state in which environmental factors, such as oxidizing agents, growth factors, cytokines, monocyte-macrophages and smooth muscle cells (SMCs), work in cooperation to promote the formation of plaque. Despite substantial progress in the prevention, diagnostics, and treatment of atherosclerosis, it remains the leading cause of mortality worldwide, approximately resulting in 18.6 million deaths in 2019 [1]. Atherosclerosis starts early in life, develops throughout a long latent and asymptomatic stage, and becomes clinically apparent only very late in the course of the disease when the lesion is already well-developed, creating areas of stenosis and/or thrombotic occlusion, reducing or totally blocking the blood and oxygen supply to the heart (coronary heart disease), the brain (ischemic stroke) or the lower extremities (peripheral vascular disease). In the last several decades, rapid advancements in understanding the pathobiologic determinants of atherosclerosis have radically changed the treatment of this disease. However, despite this, therapies designed to reverse the devastating outcomes of atherosclerosis are still limited, and current pharmacological treatments mainly aim to delay the development of the plaque, treat the symptoms and prevent the formation of blood clots. Therefore, there is an urgent need to further elucidate the cellular and molecular mechanisms involved in the appearance, progression and complications of atherosclerosis, not only to uncover the pathways contributing to the disease, but also to improve its diagnosis and management. Since we understand the molecular pathogenesis of atherosclerosis more thoroughly, new prevention and therapeutic strategies may emerge.

This Special Issue contains a selection of research articles and reviews written by experts in the field, focusing on the molecular biology of atherosclerosis, the impact of different risks factors and the mechanisms by which they can influence the development of this disease.

As briefly mentioned above, atherosclerotic disease arises as a consequence of the subendothelial retention and modification of LDL, sparking an inflammatory state and promoting the formation of fatty streaks, the earliest visible signs of atherogenesis. There is extensive evidence that increased plasma levels of proatherogenic lipid species are causally associated with atherosclerosis, and that lipid-lowering therapies can substantially reduce the lifetime risk of cardiovascular disease [2]. In this Special Issue, the role and significance of lipid accumulation during the initial stages of the disease were accurately reviewed by Torzewski, while Laudanski reported on the changes occurring in the levels of plasma lipids and lipoproteins in septic patients and their relationship with the onset of atherosclerosis, from disease initiation to the emergence of clinical complications.

Another important event contributing to the development of atherosclerosis is the recruitment of circulating monocytes to the vessel wall, their transendothelial migration and their phenotypic polarization towards proinflammatory macrophages that acquire the capability to rapidly secrete a wide range of inflammatory mediators, influencing the development and extent of atherosclerosis. Wu et al. investigated the molecular mechanisms involved in mononuclear cell (MNC) activation and endothelial dysfunction in atherosclerosis, suggesting that circulating fatty-acid-binding protein 4 (FABP4) is capable of altering monocyte cell adhesion and endothelial functions via the upregulation of ERK/JNK/STAT-1 signaling and the downregulation of the eNOS and SDF-1 pathways. Consistently, they also showed that the inhibition of FABP4 attenuates the effect of oxLDL on monocyte adhesion to ECs through the downregulation of the integrin pathways in MNCs and human coronary endothelial cells (HCAECs). Nevertheless, the paper by Chang et al. demonstrates that the selective inhibition of the C-C chemokine ligand (CCL4), an inflammatory chemokine highly expressed in atherosclerotic patients, is able to restore the endothelial adhesive properties and reduce the activity of metalloproteinase-2 and -9, as well as the production of TNF-α and IL-6 in stimulated macrophages through the nuclear factor NF-κB pathway. In the context of vascular inflammation and atherosclerosis, the role of neutrophils has been underappreciated for many years; more recently, however, neutrophils have emerged as important contributors to atherosclerosis due to their ability to release neutrophil extracellular traps (NETs). In this regard, it has been demonstrated that NETs are present in the atherosclerotic plaques of both human and animal models, where they induce endothelial cell dysfunction and apoptosis, oxidative stress and the oxidation of high-density lipoproteins; thus, reducing their capability to promote cholesterol efflux from foam cells [3,4]. Da Silva and associates investigated the expression of NET elements in human carotid plaques, demonstrating their differential expression in the upstream and downstream shoulder regions known to be subjected to different hemodynamic forces, particularly that pathological features related to plaque vulnerability (more pronounced in the downstream regions) are further associated with a higher content of neutrophils and an upregulation of NET markers (neutrophil elastase and citrullinated histone-3).

The health benefits of acetylsalicylic acid (ASA) and diminazene aceturate (DIZE) have also been investigated with respect to the atherosclerotic plaque development and phenotype. Acetylsalicylic acid (ASA), also known as aspirin, was first successfully synthesized in 1899 and widely used to reduce pain, fever and inflammation until the 1970s, when it became the cornerstone of therapy for the secondary prevention of cardiovascular events by the virtue of its antithrombotic properties [5,6]. In their work, Roth and co-workers provided interesting details regarding the protective effects of low-dose ASA on vessel wall remodeling, atherosclerosis and cardiovascular complications. By employing two different types of murine models, Apolipoprotein E-deficient (ApoE−/−) mice (the most popular murine model used for atherosclerotic studies) and ApoE−/− mice harboring a heterozygous mutation in the fibrillin-1 gene, they demonstrated that ASA treatment significantly reduced passive wall stiffness, systolic BP and cardiac remodeling. In addition, the authors suggested that ASA is able to normalize the neutrophil/lymphocyte ratio (NLR), which is an important biomarker of systemic inflammation associated with an increased risk of adverse cardiovascular events. Diminazene aceturate (DIZE) is an antiprotozoal and antitrypanosomal compound reported to enhance the catalytic activity of the angiotensin-converting enzyme 2 (ACE2) and increase the production of Angiotensin-(1–7); therefore, exerting a protective role against the adverse remodeling and dysfunction of the infarcted myocardium. Stachowicz and colleagues demonstrated that DIZE also exerts atheroprotective effects in apoE−/− mice, enhances plaque stability and attenuates hepatic steatosis by influencing macrophage polarization and taurine biosynthesis. There are other compounds that, by virtue of their anti-inflammatory and antioxidant properties, can be considered as potential therapeutic agents for atherosclerosis. Melhem and Taleb provided an overview of the fundamental aspects of tryptophan metabolism in vascular homeostasis, while Tsopka and Hadjipavlou-Litina extensively reviewed the chemical structure of the cinnamic acid–nitric oxide (NO) donor hybrids, so that the scientific community can pay more attention to their biological activities.

The review by Giglio et al. provided a clear overview of the molecular effects of novel nutraceuticals, hypolipidemic agents and hypoglycemic drugs on atherogenesis. Siew and colleagues provided valuable insight into the advances, applications and potential pitfalls of the CRISPR/Cas9 system in the context of atherosclerosis. An interesting interaction between the endothelial cells of coronary arteries (CA) and internal thoracic arteries (ITA) upon coronary artery bypass grafting (CABG) surgery was proposed by Shishkova et al. to explain the molecular mechanism underlying the increased resistance of ITA grafts to atherosclerosis and restenosis.
